# Unique combination of clinical features in a large cohort of 100 patients with retinitis pigmentosa caused by *FAM161A* mutations

**DOI:** 10.1038/s41598-020-72028-0

**Published:** 2020-09-16

**Authors:** Avigail Beryozkin, Samer Khateb, Carlos Alberto Idrobo-Robalino, Muhammad Imran Khan, Frans P. M. Cremers, Alexey Obolensky, Mor Hanany, Eedy Mezer, Itay Chowers, Hadas Newman, Tamar Ben-Yosef, Dror Sharon, Eyal Banin

**Affiliations:** 1 Department of Ophthalmology, Hadassah Medical Center, Faculty of Medicine, The Hebrew University Jerusalem, Jerusalem, Israel; 2 Department of Human Genetics, Radboud University Medical Center, Nijmegen, The Netherlands; 3 Donders Institute for Brain, Cognition and Behaviour, Radboud University, Nijmegen, The Netherlands; 4 Department of Ophthalmology, Rambam Health Care Campus, Haifa, Israel; 5 Sackler Faculty of Medicine, Tel-Aviv University, Tel Aviv, Israel; 6 Department of Ophthalmology, Tel-Aviv Sourasky Medical Center, Tel Aviv, Israel; 7 Rappaport Faculty of Medicine, Technion-Israel Institute of Technology, Haifa, Israel

**Keywords:** Genetics, Diseases, Medical research, Signs and symptoms

## Abstract

*FAM161A* mutations are the most common cause of autosomal recessive retinitis pigmentosa in the Israeli-Jewish population. We aimed to characterize the spectrum of *FAM161A*-associated phenotypes and identify characteristic clinical features. We identified 114 bi-allelic *FAM161A* patients and obtained clinical records of 100 of these patients. The most frequent initial symptom was night blindness. Best-corrected visual acuity was largely preserved through the first three decades of life and severely deteriorated during the 4th–5th decades. Most patients manifest moderate-high myopia. Visual fields were markedly constricted from early ages, but maintained for decades. Bone spicule-like pigmentary changes appeared relatively late, accompanied by nummular pigmentation. Full-field electroretinography responses were usually non-detectable at first testing. Fundus autofluorescence showed a hyper-autofluorescent ring around the fovea in all patients already at young ages. Macular ocular coherence tomography showed relative preservation of the outer nuclear layer and ellipsoid zone in the fovea, and frank cystoid macular changes were very rare. Interestingly, patients with a homozygous nonsense mutation manifest somewhat more severe disease. Our clinical analysis is one of the largest ever reported for RP caused by a single gene allowing identification of characteristic clinical features and may be relevant for future application of novel therapies.

## Introduction

Retinitis pigmentosa [RP (MIM #268000)] is the most prevalent hereditary degeneration of the retina in humans, with a prevalence of 1:4,500 (in Europe and USA)^1–4^, and 1:2,100 in the vicinity of Jerusalem^5^. RP is genetically and clinically heterogeneous, and is characterized by night blindness, progressive degeneration of photoreceptors leading to gradual loss of peripheral and then central vision, and eventually often leads to blindness^6^. On fundus examination patients typically show waxy pallor of the optic discs, attenuation of retinal vessels, and bone spicule-like pigmentary (BSP) changes, and on functional full field electroretinographic (ERG) testing responses are severely diminished and may be non-detectable^6,7^. Genetically, RP can be inherited in all Mendelian modes including autosomal recessive (AR, ~ 30% of patients), autosomal dominant (AD, ~ 20%), X-linked (~ 10%), and the remaining 40% are isolate cases^8^.

Currently, mutations in 41 genes were reported to cause non-syndromic ARRP (RetNet, https://sph.uth.edu/retnet/), including *FAM161A*, which has been identified in 2010 simultaneously by others and by us^9,10^. *FAM161A* was found to be localized to the base of the connecting cilium, the basal body region, and the adjacent centriole in photoreceptor cells^11–13^, and is part of the cytoskeleton fraction of the cilia and a component of the human centrosome^14,15^. It was also found to be a member of the Golgi-centrosomal interactome, a network of proteins interconnecting Golgi maintenance, intracellular transport and centrosome organization^16^.

**Figure 1:**
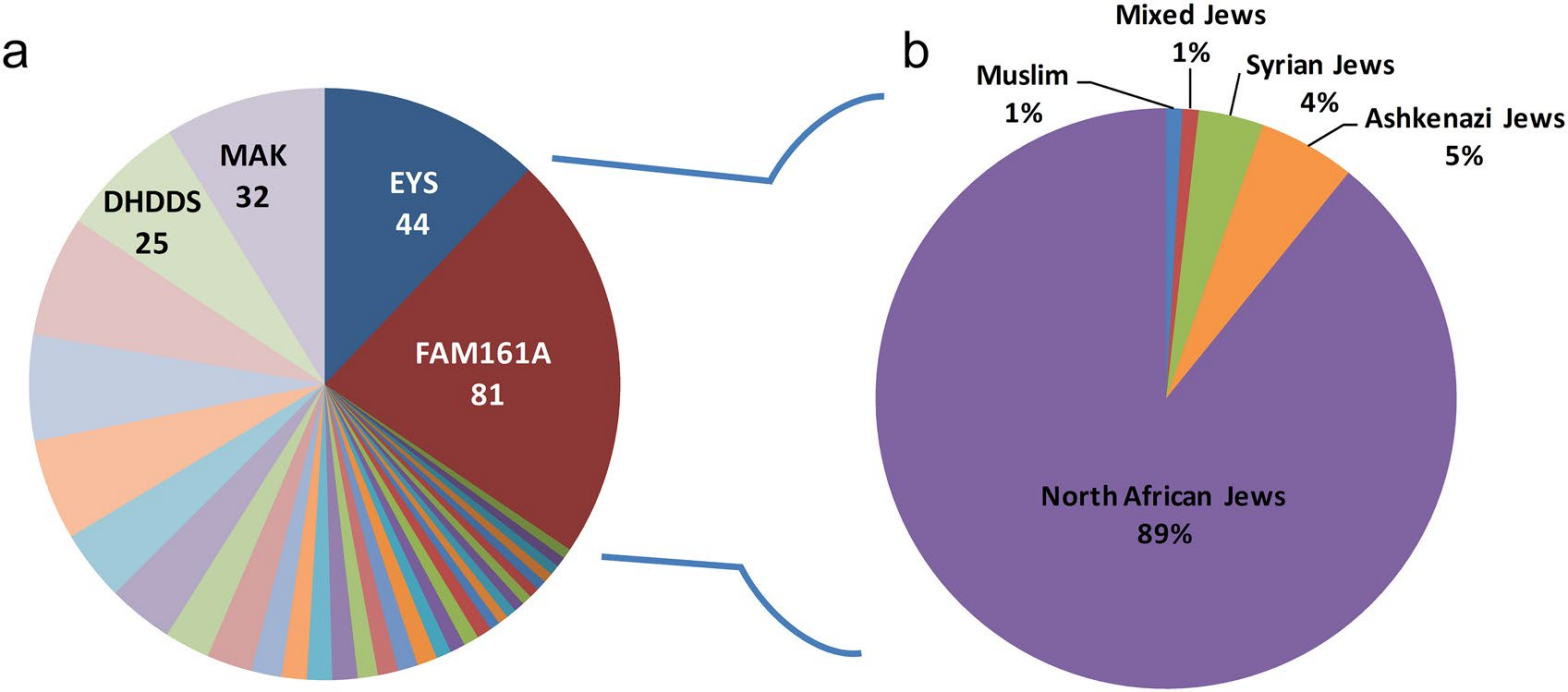
Distribution of ARRP causative genes and ethnic origin of *FAM161A* families in the cohort of Israeli patients. (**a**) *FAM161A* mutations are the major cause of ARRP in the Israeli population, responsible for ~ 20% of the families with ARRP in our cohort in which the causative gene was identified. Genes that were found in more than 25 families are listed by name and the number of families affected is indicated. (**b**) Ethnic origin of the 81 *FAM161A* families in the Israeli cohort. The vast majority are of North African Jewish descent.

Since the identification of *FAM161A* as a cause for ARRP in 2010, 13 pathogenic mutations have been reported (Supplementary Fig. S1 and Supplementary Table S1)^9,10,17–27^. Two of these mutations (frameshift c.1355_6del and nonsense c.1567C > T) were originally identified to be relatively common among the Jewish population in Israel^9^ and two were reported in Palestinian families^9,20^. Mutations in this gene are the most common cause of ARRP in Israel (18.2%, Fig. 1a) while the frequency elsewhere is relatively low (ranging from 0.003 to 2%^17,19,21,22,28^).

To date, a total of 82 patients with ARRP caused by *FAM161A* mutations have been reported in various publications (42 residing in Israel and 53 worldwide), with a phenotype that largely falls within the spectrum described in other genes causing ARRP^9,10,17–22,28^. In the current study we further explore the clinical phenotype in a large cohort of 100 Israeli patients harboring *FAM161A* mutations for whom clinical data was available. This aims to provide information on the clinical spectrum and course of disease associated with this gene, including identification of characteristic features. The findings can assist in the evaluation and diagnosis of these patients, provide data on prognosis, and may be relevant for future application of novel therapies such as gene augmentation, including windows for intervention and possible outcome measures.

## Results

### Identification of *FAM161A* mutations

We analyzed more than 1,500 index cases from our cohort, who suffer from AR or isolate RP, or other IRD types. Using a number of molecular genetic methods, as detailed above, biallelic *FAM161A* mutations were identified in 81 index cases, with a total of 114 patients in these 81 families-42 of whom were previously reported^9^, with emphasis on the genetic findings (Supplementary Table S2).

The vast majority of mutated alleles were found among Jewish patients with an RP phenotype (113 patients from 80 families). Among Jewish families with non-syndromic ARRP in our cohort, *FAM161A* is the leading genetic cause, responsible for 18.2% of families. Biallelic *FAM161A* mutations were found in only one Muslim family with ARRP, as we previously reported^9^. None of the families with other IRD phenotypes was found to carry *FAM161A* mutations, indicating a rather restricted clinical spectrum associated with *FAM161A* mutations. Out of the 114 patients, 100 are homozygous for the disease-causing mutation and 14 are compound heterozygotes.

### Clinical analysis of *FAM161A* patients

Clinical data of 100 *FAM161A* patients were available, ranging in age between 3.5 and 86 years old (Supplementary Table S2). No consistent involvement of organs other than the eye was identified in this large cohort of patients, suggesting that *FAM161A* is associated only with an ocular phenotype and is not a cause of syndromic RP.

The most frequent initial symptom was night blindness, followed by loss of visual fields and decrease in visual acuity. Most of the patients reported that the initial symptom of night blindness appeared in childhood (39 patients before the age of 10) or during adolescence (26 patients between the ages of 11–20), which represents 78% of the 83 patients for whom this data was available (Supplementary Table S2).

### Visual acuity (VA) and refractive error

Data on the refractive error was available for 63 patients, with all showing myopia ranging from − 0.75 to − 15.0 D. The majority manifest moderate to high myopia, as reflected in the mean error being − 6.2 D ± 2.93 (Supplementary Table S2).

**Figure 2:**
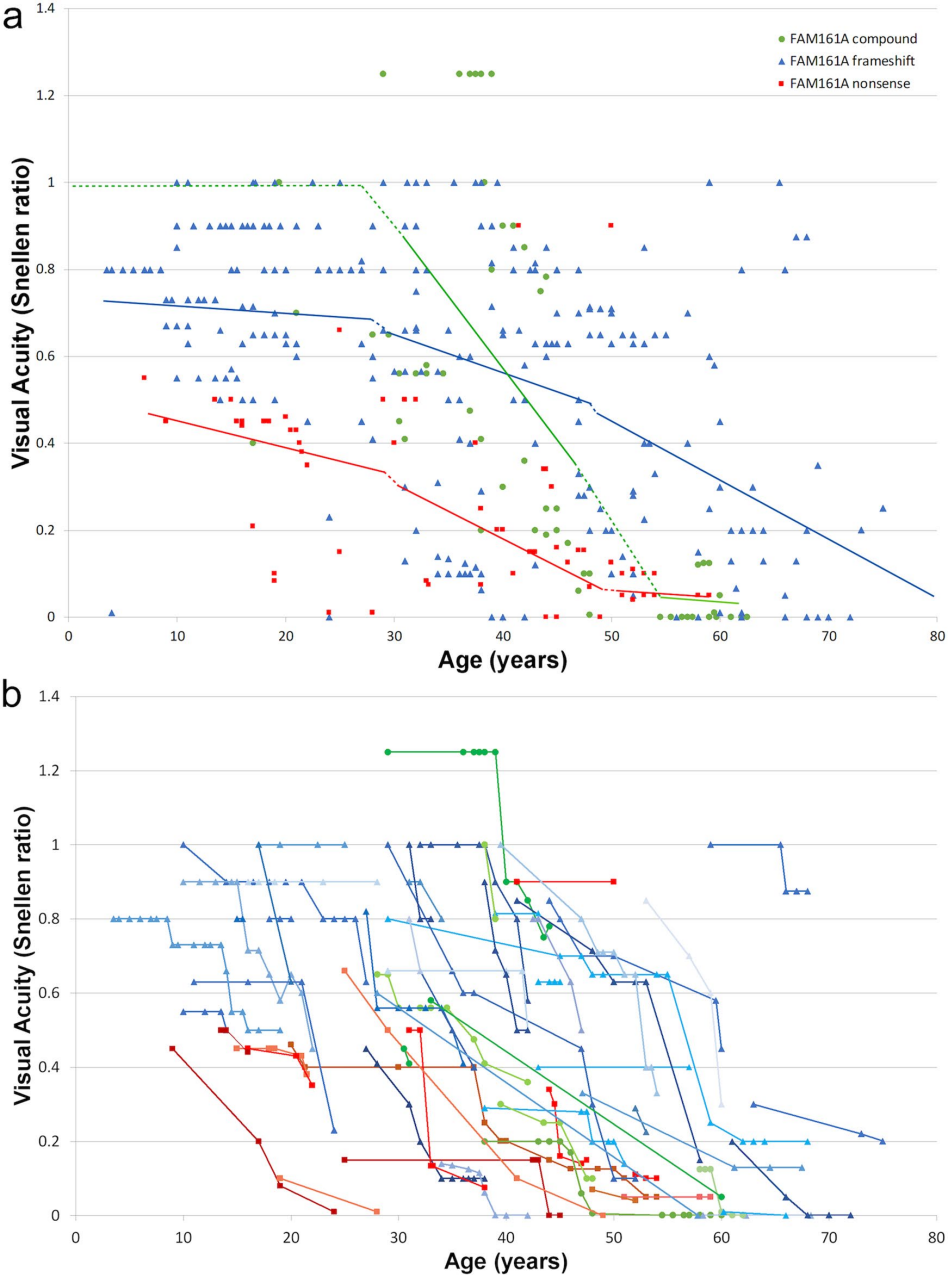
Visual Acuity of *FAM161A* patients at different ages. (**a**) Distribution plot of 366 best-corrected visual acuity (VA) measurements in 92 *FAM161A* patients at different ages. Each point represents the average VA between the two eyes at the time of each visit, computed as a Snellen fraction. Data was divided into 3 groups based on the genotype, each symbolized by a different color and shape. Linear regression analysis for the three different genotypes, with separate analysis for each of three age groups (younger than 30, between 30–50, and older than 50 years of age), is shown by the colored lines. The dotted lines are extrapolations to connect the different age groups. (**b**) Serial Snellen ratio VA levels in 63 patients for whom at least 2 measurements were available. Each patient is shown by a different colored line, with nonsense mutation genotype patients depicted in reddish hues, patients homozygous for the frameshift mutation shown in different shades of blue and compound heterozygotes in green colors.

Best-corrected visual acuity (BCVA) was measured using ETDRS charts, and is presented using Snellen values (Fig. 2a; all the analyses detailed below were also performed on BCVA expressed in LogMAR and yielded similar results, hence only Snellen equivalents are reported). BCVA data in at least one visit was available in a total of 92 patients, and in 63 of them additional measurements were available. A total of 366 BCVA measurements were made between the ages of 3.5–86 years with follow-up periods ranging from 6 months to 57 years. In the majority of patients, until the age of 30, VA seems to be relatively preserved and stable, with a mean of 0.66 ± 0.26 (SD). Between the ages of 30 to 65 progressive loss of central acuity occurs, and most patients deteriorate to very poor BCVA after the age of 50 (mean BCVA 0.25 ± 0.29 in this subgroup). Analysis according to *FAM161A* genotype suggests that patients harboring the common nonsense mutation in the homozygous state show earlier loss of BCVA as compared to patients with homozygous frameshift or compound heterozygote mutations (Fig. 2a and Supplementary Table S3) and patients who are compound heterozygous seem to lose their VA faster than patients with the common frameshift mutation. Analysis of covariance for average Snellen acuity revealed that both age and genotype independently and together affect VA in a highly statistically significant manner (*p* < 0.001, Supplementary Table S3). Regression analysis for the three different genotypes, with separate analysis for each of three age groups (younger than 30, between 30 and 50, and older than 50 years of age), is shown in Fig. 2a. Rate of VA loss between the ages of 30–50 is similar between patients with the homozygous nonsense and frameshift mutations with the nonsense mutation patients usually starting and ending with lower VAs, and the rate of decline may be higher in patients with the compound heterozygous genotype (Fig. 2a). The extensive dataset with up to 19 serial BCVA assessments in a given patient and follow-up ranging from 6 months to 57 years allowed to also examine BCVA over time in a significant number of patients. Thus, serial follow-up in 63 patients (Fig. 2b) showed similar trends to those described above: *FAM161A* patients usually presented in their second to third decades of life with mildly reduced VA, and showed marked loss by the sixth decade, with patients homozygous for the nonsense mutation manifesting lower BCVA at younger ages.

Cataract was rare before the age of 30, with slowly progressive posterior subcapsular opacity developing from the fourth decade and on. Cataracts were identified in 53 out of the 74 patients (71%) for which data on examination of the lens was available but these were usually mild, with only 15 of these patients requiring cataract surgery (mean age at surgery being 47.5 years, range 21–68).

### Fundus findings

**Figure 3:**
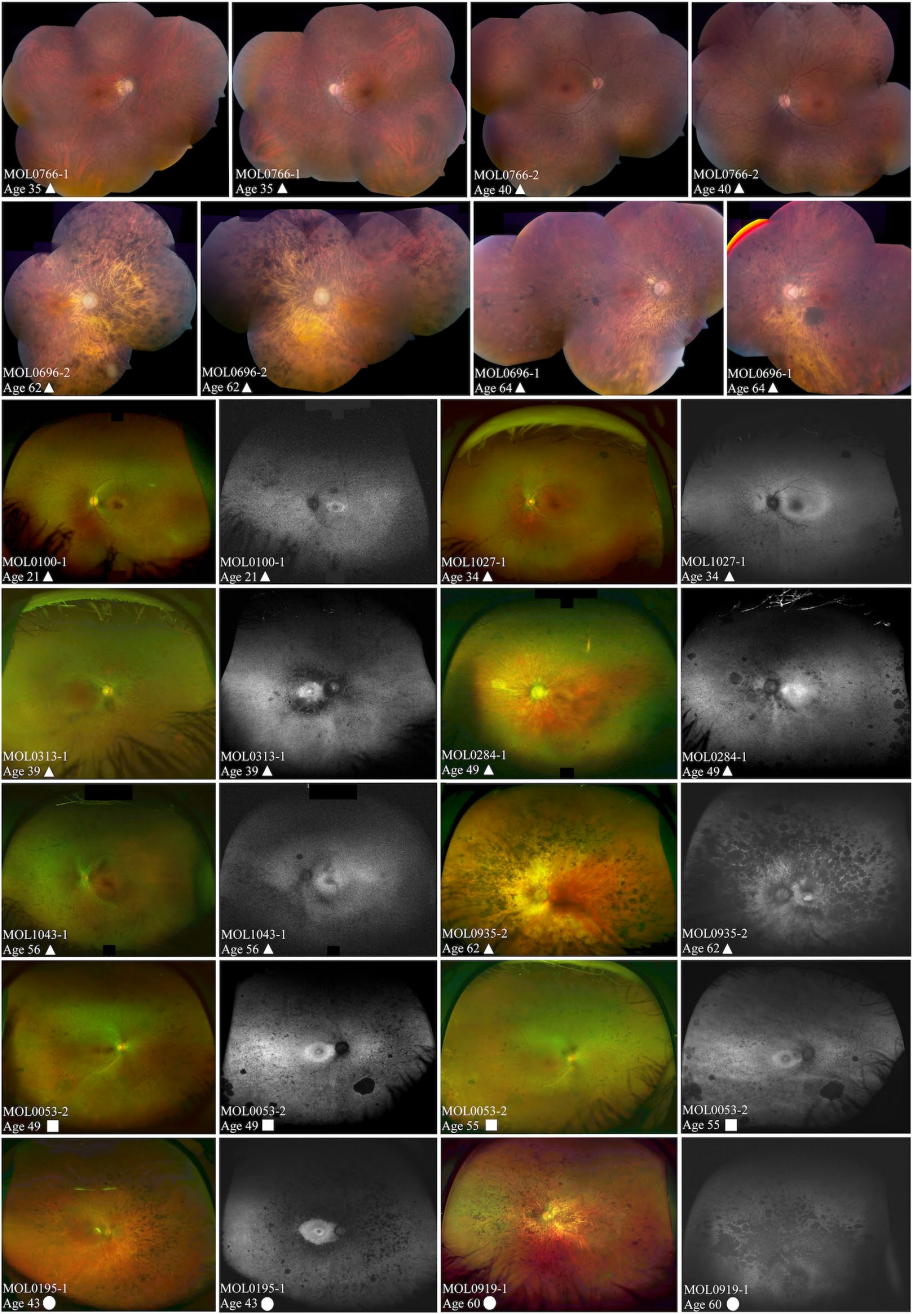
Spectrum of fundus findings among different *FAM161A* patients at different ages. Color fundus mosaics (top 2 rows) and wide field OPTOS pseudocolor and fundus autofluorescence (FAF) images (bottom 5 rows) show that while the degenerative changes in FAM161A patients largely fall within the spectrum generally associated with RP, bone spicule-like pigmentary changes are relatively mild in most patients up to 40–50 years of age. In addition, in older patients (ages 50 +), nummular pigmentation was observed. Patient numbers, age at time of imaging and genotype are specified on each of the panels (triangles represent homozygousity for the frameshift mutation, squares homozygosity for the nonsense mutation and circles denote compound heterozygous patients).

Although fundus findings in patients with *FAM161A* mutations largely fall within the spectrum generally observed in RP, there are some unique features characteristic of these patients. Waxy pallor of the optic discs and attenuation of retinal vessels appear at relatively early ages, but bone spicule-like pigmentary changes (BSP) appear late in life, with initial pigmentation often observed in the mid-periphery only after the age of 30. These changes are accompanied by retinal atrophy. BSP remains relatively mild in most patients up to 40–50 years of age. In addition, in older patients (ages 50 +), nummular pigmentation (patches of irregular pigmentary deposits) is observed. Color and fundus autofluoresence photos (FAF) were available for 76 patients and representative images of 10 of the patients at various ages and disease stages are presented in Fig. 3. FAF images showed a hyper-autofluorescent ring around the fovea in the vast majority of patients, including at young ages (second decade of life), along with hypo-autofluorescent spots in the mid periphery (Fig. 3).

### Ocular coherence tomography (OCT)

**Figure 4:**
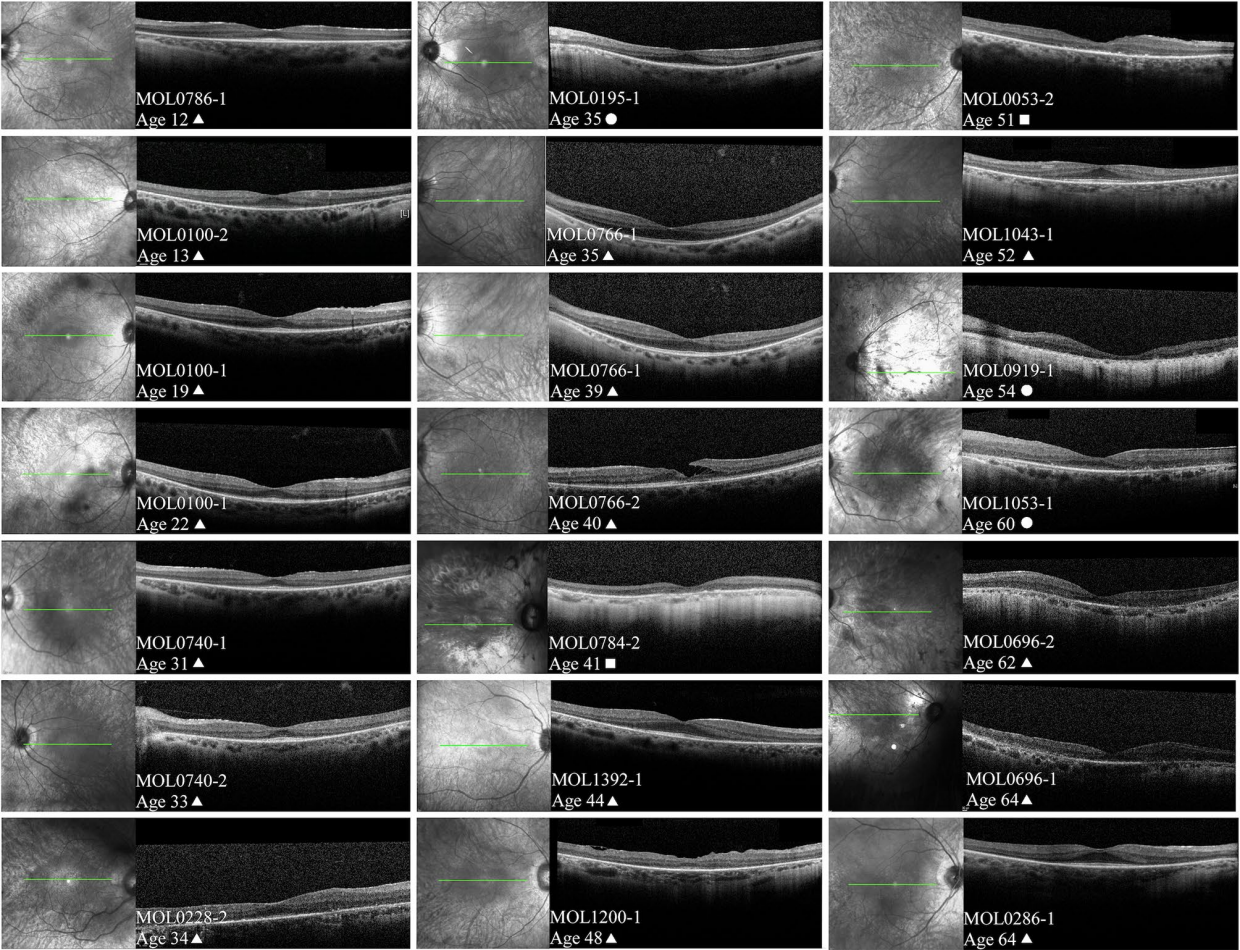
Representative OCT (ocular coherence tomography) images. Infrared imaging of the macular area and OCT scans passing through the fovea from 19 patients with *FAM161A*-related retinal degeneration are shown, arranged according to patient age. In two patients, OCT scans that are 3 years (MOL0100-1) and 4 years (MOL0766-1) apart are presented. In general, increased thinning of the photoreceptor layer and loss of intact ellipsoid zone width occurs with age, with relative preservation in the foveal area. Above the age of 50, progression to foveal atrophy can be observed in some cases (for example, patient MOL0919-1). Interestingly, cystoid macular changes seem to be quite rare in *FAM161A* patients. Patient numbers, age at time of imaging and genotype are specified on each of the panels (triangles represent homozygousity for the frameshift mutation, squares homozygosity for the nonsense mutation and circles denote compound heterozygous patients).

OCT imaging was available for 82 patients and foveal scans from 19 of them are presented according to patient age in Fig. 4. In two patients, OCT scans 3 years (MOL0100-1) and 4 years (MOL0766-1) apart are shown. In general, increased thinning of the photoreceptor layer (outer nuclear layer, ONL) occurs with age, with relative preservation of the ONL and ellipsoid zone (EZ) in the foveal area. Towards and above the age of 50, only a thin layer of photoreceptors is seen in the fovea, there is loss of the EZ, and in some cases this progresses to foveal atrophy (for example, patient MOL0919-1). In many cases (74 out of 82 patients) thickening of the inner limiting membrane or a fine epiretinal membrane (ERM) was observed in at least one eye, although often not in the foveal area, and not to the degree that necessitates surgical intervention. A lamellar macular hole was noted in one eye of MOL0766-2 at the age of 40. Interestingly, cystoid foveal and macular changes seem to be quite rare in *FAM161A* patients: small intraretinal cysts were identified in only 4 out of the 82 patients for whom OCT imaging was available, and frank cystoid changes were not observed at all.

### Visual fields

**Figure 5:**
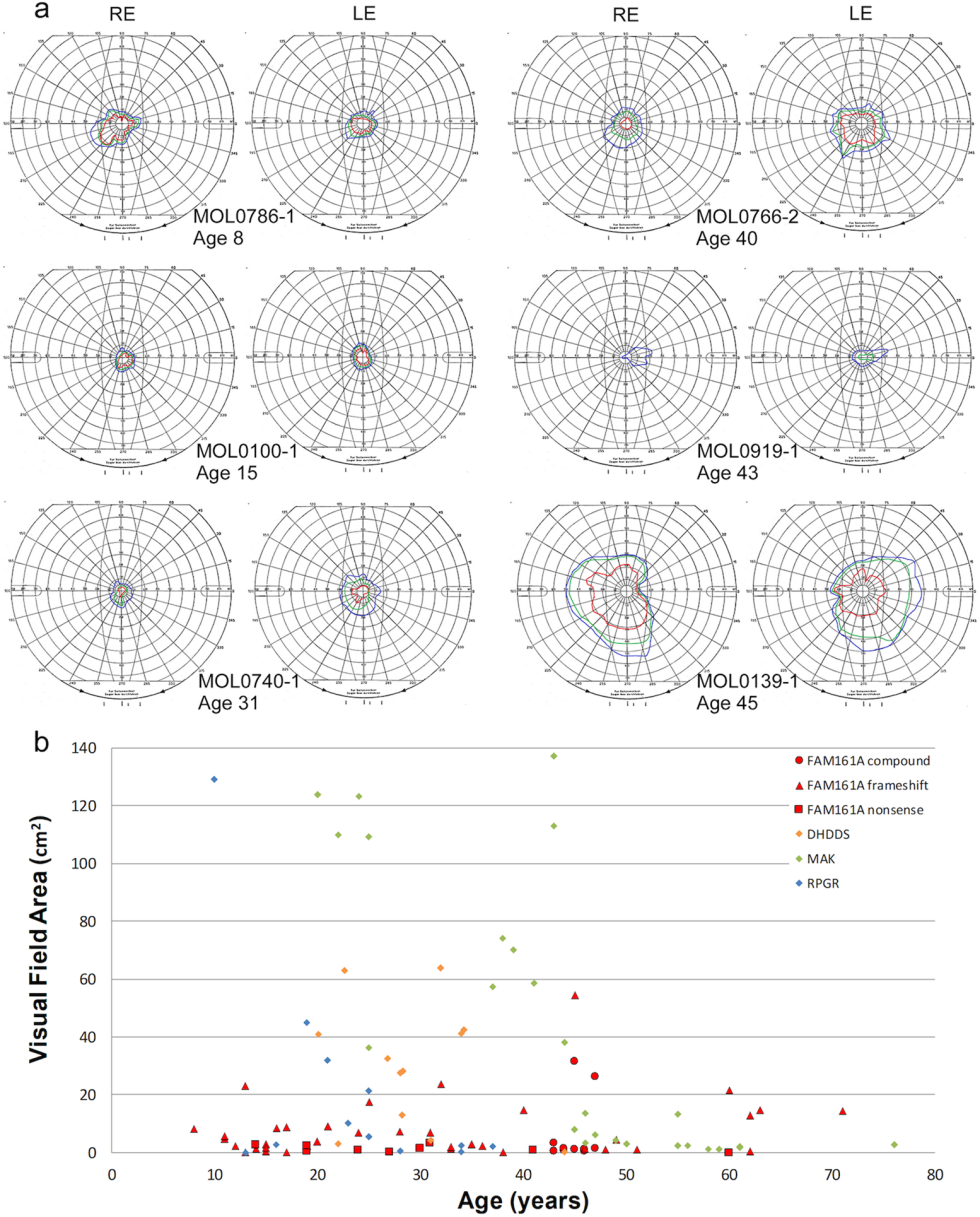
Goldmann kinetic perimetry in patients with biallelic *FAM161A* mutations. (**a**) Examples of visual fields from 6 patients at different ages show that constriction of the fields occurs relatively early in the majority of FAM161A patients, although occasionally fields may be better preserved into the fourth or even fifth decade. Target I4e shown in red, target III4e in green and target V4e in blue. (**b**) Visual field area (using target V4e) plotted versus age in patients with *FAM161A* mutations as compared to other causative genes in our cohort. Visual field areas from 50 *FAM161A* patients and 39 patients with other RP-causing genes (*DHDDS* [blue], *MAK* [red] and *RPGR* [green]- males only) are presented. Each symbol represents the average between the two eyes. Only genes with data from at least 10 patients were included.

Examples of Goldmann kinetic visual fields of six *FAM161A* patients at different ages are presented in Fig. 5a. In the majority of cases marked constriction even for the large V4e target is evident from an early age (note fields of patient MOL0786-1 at age 8 and patient MOL0100-1 at age 15). Interestingly, many patients reported being identified as "clumsy" in their childhood and teen years, probably reflecting such early visual field constriction. Rarely, relatively well preserved fields can be found in patients even beyond the age of 40 (for example, patient MOL0139-1, Fig. 5). We were able to collect visual field data from 50 patients with *FAM161A* mutations and 39 patients with RP due to other genes (Fig. 5b). In the vast majority of *FAM161A* patients (49/50), as described above, visual fields are constricted, even at relatively young ages. Patients with mutations in *RPGR* and *DHDDS* are also usually symptomatic and seek medical attention early in life, but seem to do somewhat better than *FAM161A* patients in this regard, still maintaining sizeable fields up to their early twenties. Patients with mutations in *MAK* are usually diagnosed later in life and visual fields are also affected much later. Significant differences were found between *FAM161A* patients as a group and patients with RP due to *MAK* (*p* < 0.001), *RPGR* (*p* < 0.001) and DHDDS mutations (*p* = 0.005), with *FAM161A* patients showing more severe constriction of visual fields at earlier ages (Supplementary Table S4). In summary, in our cohort, *FAM161A* patients as a group tend to present through all ages with severely constricted visual fields.

### Electroretinography (ERG)

**Figure 6:**
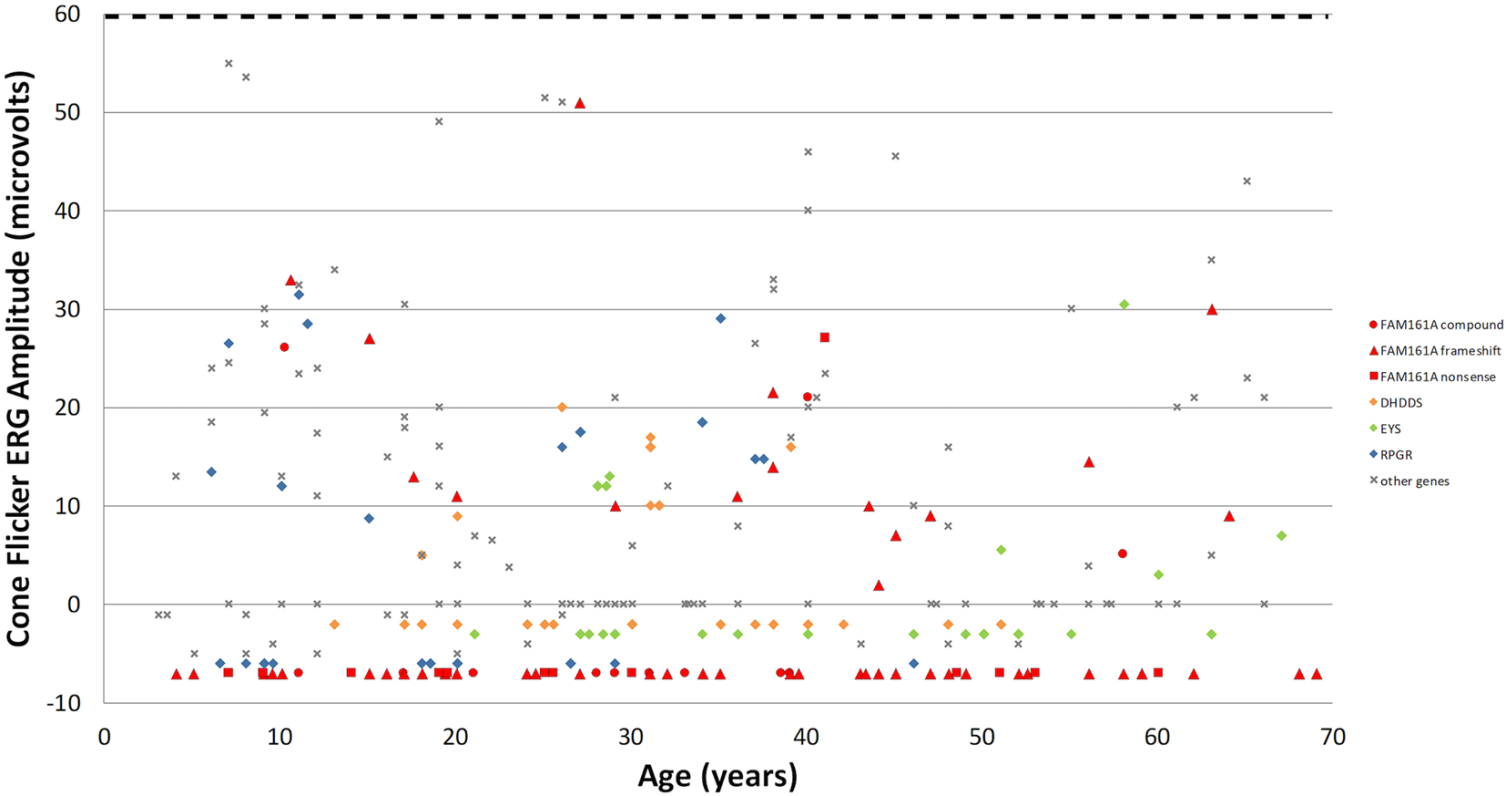
Cone flicker ERG amplitudes at 1st testing. A total of 231 genetically identified patients were included, and the average cone flicker amplitude between the two eyes as measured at the earliest ERG test performed in each of the patients is presented. Genes with at least 20 patients each (*FAM161A* [red, with different shapes representing the three genotypes)], *DHDDS* [orange diamond], *EYS* [green diamond], *RPGR [blue diamond]*) are specifically marked, while genes with less than 20 patients per gene share the same annotation (gray x) on the graph (*ABCA4-* only patients with an RP phenotype, *ADAM9*, *BBS2*, *C2ORF71*, *C8ORF37*, *CDHR1*, *CERKL*, *CNGA1*, *CNGB1*, *CRB1*, *IMPG2*, *MAK*, *NRL*, *PDE6A*, *PDE6B*, *PLA2G5*, *PRPF3*, *RDH12*, *RHO*, *RP1*, *RP2*, *RPGRIP1*, *USH1C*, and *USH2A*). Since in many patients cone flicker responses were non-detectable, to allow better visualization these "zero" points were spread over the values of – 10–0 uV. Lower limit of normal for cone flicker ERG amplitudes at our lab is 60 uV, marked by a horizontal dashed line at the top of the figure.

ERG data was available for 93 patients, with mean age at first testing being 34 years old (range 7–68 years). In 81 (87%) of the cases, rod responses were already non-detectable on this first exam, and cone flicker was non-detectable in 67 (72%) of them (Fig. 6 and Supplementary Table S2). The relatively late mean age of first ERG testing seems to correlate with the age range at which visual acuity deteriorates in the majority of patients (as described above and shown in Fig. 2a) and with the relative preservation of ONL in the foveal area up to these ages. However, by this time, ffERG rod as well as cone function are already non-detectable in over 70% of patients which seems to correlate with the relatively early loss of night vision and marked constriction of visual fields observed in many of the *FAM161A* patients. Looking at cone flicker responses among the three different genotypes of *FAM161A*, 1/15 (7%) of patients homozygous for the nonsense mutation had detectable cone flicker responses at time of first ERG recording, versus 16/64 (25%) of patients homozygous for the frameshift mutation and 3/14 (21%) of patients with compound mutations. Although the homozygous nonsense group seems to have more severely affected cone flicker ERG responses at time of first testing, analysis using the Fisher exact test did not show a statistically significant difference, probably due to an insufficient number of patients available for analysis in this group. Cone flicker ERG amplitudes at time of first ERG recording in *FAM161A* patients as compared to other RP-causing genes are also presented in Fig. 6. Age at first testing of *FAM161A* patients is widely variable, in similarity to other genetic causes, but differing from others such as *DHDDS* and *RPGR* that rarely present after the age of 40.

## Discussion

In the current study, 114 patients with bi-allelic *FAM161A* mutations were identified, 113 of them of Jewish origin and one of Arab-Muslim descent. As far as we know, this is the largest genetically-diagnosed cohort of *FAM161A* patients worldwide. All Jewish *FAM161A* patients are either homozygous or compound heterozygous for two founder mutations (c.1567C > T and c.1355_6del; Supplementary Table S2).

Clinical findings in a small number of patients with *FAM161A*-RP have been reported previously, including by ourselves^10,17–20,28^. In the present paper, we report on the largest set of *FAM161A* patients studied thus far worldwide. This allowed us to identify a characteristic, rather unique combination of clinical features that differentiate *FAM161A*-RP from RP caused by other genes.

The initial clinical diagnosis in all patients with biallelic *FAM161A* mutations was RP. In the wide screening we performed among our cohort of patients with other clinical IRD phenotypes, none were found to carry biallelic *FAM161A* mutations. This indicates a rather narrow phenotypic spectrum associated with *FAM161A* mutations, and differs from the *ABCA4* gene, for example, which can be associated with a very wide range of clinical phenotypes ranging from the maculopathy of typical Stargardt disease to CRD and RP phenotypes^29^.

From among the 114 genetically diagnosed patients, clinical data of any kind was available in 100 patients. Availability of data for the different parameters presented ranged from 50 (Goldmann visual fields) to 93 (ERG data), as detailed above. The patients in our study demonstrated rather typical symptoms of RP, with the leading initial symptoms being night blindness and constriction of the visual fields. Rarely, loss of visual acuity was reported as the initial symptom. Most of the patients (78%) reported that these symptoms, and mainly night blindness, appeared during the first two decades of life. Visual acuity is largely preserved in *FAM161A* patients through the first 3 decades of life, and then progressively deteriorates over the next 2–3 decades. The good VA that carries into the early thirties may be related to the relatively late age (34 years on average) at time of first ERG testing, even though by then rod as well as cone responses are non-detectable in the majority of patients and visual fields are also markedly constricted. Later on, patients may experience loss to the levels of finger counting or light perception at older ages, but deterioration to the level of NLP is extremely rare (one eye in each of two different patients from our cohort). Interestingly, a small subgroup of *FAM161A* patients maintain good VA as well as relatively well preserved visual fields and cone ERG responses even up to the 6th and even 7th decade of life. At the other extreme, there are occasionally young patients presenting with rather severe disease already in their late teens/early twenties. The majority of *FAM161A* patients manifest moderate to high myopia while cataracts are usually mild and not deemed clinically significant until late in life.

Retinal imaging in patients with *FAM161A* mutations showed that BSPs are relatively mild in most patients up to 40–50 years of age, perhaps related to the underlying myopia. In addition, in older patients (ages 50 +), patches of irregular pigmentary deposits were observed. A hyperfluorescent ring surrounding the macula is often seen on FAF imaging. Delicate ERMs that were often extrafoveal were identified in 90% of the patients (even at young ages), as compared to the general RP population, where only 27.3% of the patients were identified with ERM^30^. Interestingly, cystoid macular changes were extremely rare: small intra-retinal cysts were identified in only 4 out of the 82 patients for whom OCT imaging was available, and frank cystoid changes were not observed at all. This differs from the prevalence of CME generally reported in RP, which is estimated at 10–50%^30,31^.

Our dataset includes biallelic *FAM161A* patients with three possible genotypes: homozygotes for the c.1355_6del frameshift mutation, homozygotes for the c.1567C > T nonsense mutation, and compound heterozygotes for both mutations. Interestingly, our clinical data suggests that the disease associated with homozygosity to the nonsense mutation may confer somewhat more severe disease. There is a statistically significant difference in VA between patients who are homozygous for the nonsense mutation compared to patients who are either compound heterozygotes or homozygous for the frameshift mutation, and cone flicker responses at time of first ERG testing may also be more severely affected in homozygous nonsense mutation patients. Since both mutations create a premature stop codon within exon 3, the corresponding mRNAs can either be degraded by the nonsense mediated mRNA decay (NMD) surveillance mechanism or, if the expressed abnormal mRNAs escape this mechanism, might yield mutated truncated proteins. Although no data is available on NMD activity on *FAM161A* mutations in the human retina, studies in human cell lines show that *FAM161A* aberrant transcripts can indeed escape this mechanism. Assuming that a similar scenario takes place in the human retina, one would expect two possible outcomes: the truncated protein is unstable and degraded, or it might be expressed yielding either partially active protein, nonfunctional protein, or a toxic one. The nonsense mutation possibly produces a toxic protein while the shorter protein produced by the frameshift mutation is either non-toxic or is less toxic to photoreceptors. Additional studies are needed to shed light on this complicated issue; however, since results might differ between tissues and species, studies of retinal human samples with these mutations (which are currently unavailable) will be required.

Our cohort of IRD patients includes 704 index cases with nonsyndromic RP. The causative gene is currently identified in 387 (55%) of the families, with *FAM161A* being the major one. Among the families with ARRP, *FAM161A* mutations account for a large fraction of approximately 20%. Together with three additional genes (*EYS*, *DHDDS* and *MAK*), this account for almost 50% of ARRP cases in our cohort (Fig. 1a). Among Jewish *FAM161A* patients (that comprise 99% of this cohort), the vast majority (89%) are of North African descent (Fig. 1b). Specifically looking at this subgroup of North African Jewish (NAJ) patients with ARRP, *FAM161A* accounts for about half of the cases.

**Figure 7:**
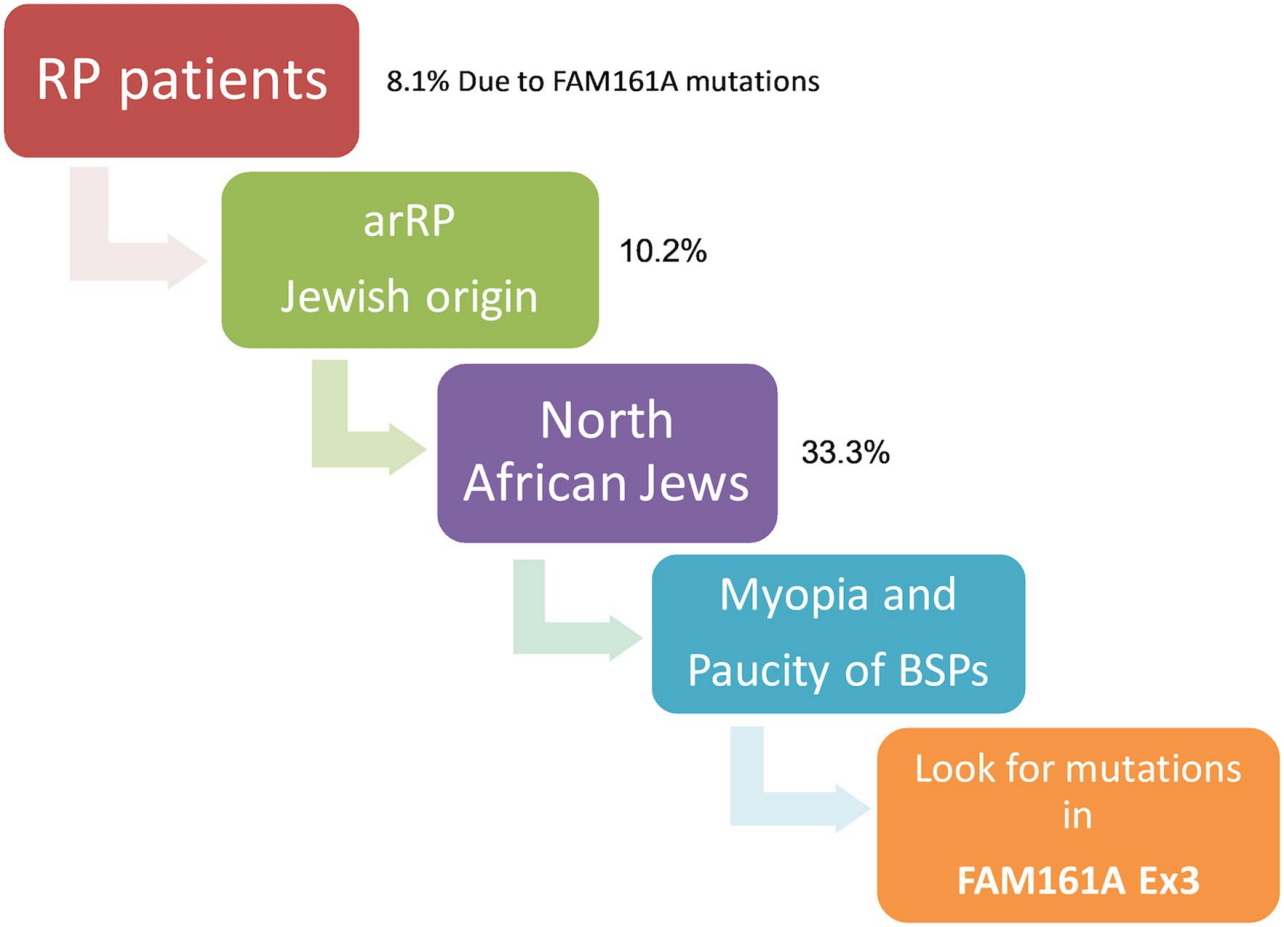
Flow chart of conclusions with practical implications.

The findings detailed above thus allow drawing of conclusions with practical implications: in our population, if a Jewish patient presents with RP that is thought to be AR per family history or is an isolate case, there is a ~ 10% chance that *FAM161A* is the causative gene. If the patient is of NAJ origin, the chances further increase to ~ 33%. If, however, the patient is of Arab Muslim descent, the chance is less than 0.01% (Fig. 7 and Supplementary Table S2). Clinical features that may further support *FAM161A* as the causative gene include moderate-high myopia, constricted VFs at early ages but with relatively well preserved VA, and paucity of BSP-like pigmentary changes. The presence of frank, severe CME or of advanced cataractous changes may decrease the probability that *FAM161A* is the underlying cause of disease.

In conclusion, we have identified 114 patients, 113 of them from Jewish origin, with *FAM161A*—related RP. The genetic and clinical findings in the cohort described here may provide better understanding of the manifestations and progression of this disease, and may also serve to guide and assess the effects of future therapeutic interventions, including gene augmentation therapy.

## Methods

All methods were carried out in accordance with relevant guidelines and regulations.

### Subjects

We recruited for the study Israeli and Palestinian individuals with various inherited retinal dystrophies (IRDs). Before drawing a blood sample for molecular analysis, all participants in the study signed an informed consent that adhered to the tenets of the declaration of Helsinki. Ethical approval for this study was obtained from the Hadassah-Hebrew University Medical Center and the Rambam Health Care Center IRB committees.

### Genetic analysis

Genetic analysis was performed in all cases using either homozygosity mapping^9^, Sanger sequencing based on ethnicity-matched known mutations, whole exome sequencing (WES)^32^, or molecular inverted probes (MIPs) analysis^33^. Primers for all suspected variants were designed using the Primer3 online program (https://www.bioinformatics.nl/cgi-bin/primer3plus/primer3plus.cgi/) (Supplementary Table S5). In all patients, independent of the initial method used to identify the causative mutation/s, Sanger sequencing of PCR products was performed to verify the result.

### Clinical evaluation

Clinical data was collected retrospectively at one center (Hadassah-Hebrew University medical center) from the medical records of 100 patients harboring biallelic *FAM161A* pathogenic mutations. Briefly, clinical information included, when available, the following: anamnestic information on disease onset, progression and symptoms, best-corrected visual acuity (BCVA), refractive error, clinical ocular exam by slit lamp biomicroscopy, full-field electroretinography (FFERG), Goldmann visual fields (using the I4e, III4e and/or V4e targets according to stage of disease), ocular coherence tomography (OCT, using the Heidelberg Spectralis system), color, infrared and fundus autofluorescence (FAF) imaging (using a Zeiss and/or Optos fundus camera and the Heidelberg Spectralis system).

Full-field ERG responses were recorded according to the ISCEV standard using corneal electrodes and a computerized system (UTAS 3,000, LKC, MD) as previously described^34^. Briefly, in the dark-adapted state, a rod response to a dim blue flash and a mixed cone-rod response to a white flash were acquired. Cone responses to 30-Hz flashes of white light were acquired under a background light of 21 cd/m^2^. All responses were filtered at 0.3–500 Hz and signal averaging was used. For assessment of severity of disease as compared to other RP-causing genes in our cohort, cone 30 Hz flicker amplitudes were measured in 231 genetically identified patients with RP. The average cone flicker amplitude between the two eyes as measured on the earliest ERG test performed in each of the patients was included in our analysis (age at first ERG testing may be indicative of disease severity).

BCVA was measured at each visit of the patient and the average of both eyes was taken. In case the patient underwent cataract surgery and the BCVA improved in the operated eye, measurements prior to surgery that were lower in this eye were corrected to the measurement post-surgery with the thought that this better represents retinal function at that time. In order to provide numerical values for low BCVAs, the following conversions were made: NLP (no light perception) = 0, LP (light perception) = 0.0001, HM (hand movement) = 0.001, FC (finger counting) = 0.01^35^. When necessary, LogMAR of BCVA was converted to the Snellen equivalent using an online converter program (www.myvisiontest.com/logmar.php).

### Statistical analysis

The ANCOVA model was applied to VA data to assess the effect of age, mutation and the interaction between mutation and age on VA, for all measurements of VA without taking into account the patient's effect. In addition, a repeated measures model using Generalized Estimating Equations was applied for testing the effect of age, mutation and the interaction between mutation and age on VA. This model tests the significance of these effects while taking into account the dependence between VA measurements from the same patient, where patients can each have a different number of repeated measurements. A similar analysis was performed on VF data comparing FAM161A to other RP-causing genes (DHDDS, RPGR, and MAK).

All tests applied were two-tailed and a *p* value of 0.05 or less was considered statistically significant.

## Supplementary information

Supplementary Information.

## Data Availability

The datasets generated and/or analyzed in the current study are available from the corresponding author upon reasonable request.
