# Supplementary material for: Unique combination of clinical features in a large cohort of 100 patients with retinitis pigmentosa caused by FAM161A mutations
Source: Sci Rep. 2020 Sep 16;10:15156. doi: 10.1038/s41598-020-72028-0 (PMC7495424; doi:10.1038/s41598-020-72028-0)
Supplement: Supplementary file 1 — Supplementary Information. [file 41598_2020_72028_MOESM1_ESM.pdf]

Unique Combination of Clinical Features in a Large Cohort of 100 Patients With Retinitis Pigmentosa Caused by *FAM161A* Mutations

Avigail Beryozkin[1], Samer Khateb[1], Carlos Alberto Idrobo-Robalino [1], Muhammad Imran Khan[2], Frans Cremers[2,3], Alexey Obolensky[1], Mor Hanany[1], Eedy Mezer[4], Itay Chowers[1], Hadas Newman[5, 6], Tamar Ben-Yosef[7], Dror Sharon[1]*, and Eyal Banin[1]*

[1] Department of Ophthalmology, Hadassah-Hebrew University Medical Center, Jerusalem, Israel.

[2] Department of Human Genetics, Radboud University Medical Center, Nijmegen, The Netherlands.

[3] Donders Institute for Brain, Cognition and Behaviour, Radboud University, Nijmegen, The Netherlands.

[4] Alberto Moscona Department of Ophthalmology, Rambam Health Care Campus, Haifa, Israel.

[5] Sackler Faculty of Medicine, Tel-Aviv University, Tel-Aviv, Israel.

[6] Department of Ophthalmology, Tel-Aviv Sourasky Medical Center, Tel-Aviv, Israel.

[7] Rappaport Faculty of Medicine, Technion-Israel Institute of Technology, Haifa, Israel.

Supplementary Figure 1: Schematic representation of the *FAM161A* pathogenic mutations that have been reported previously.

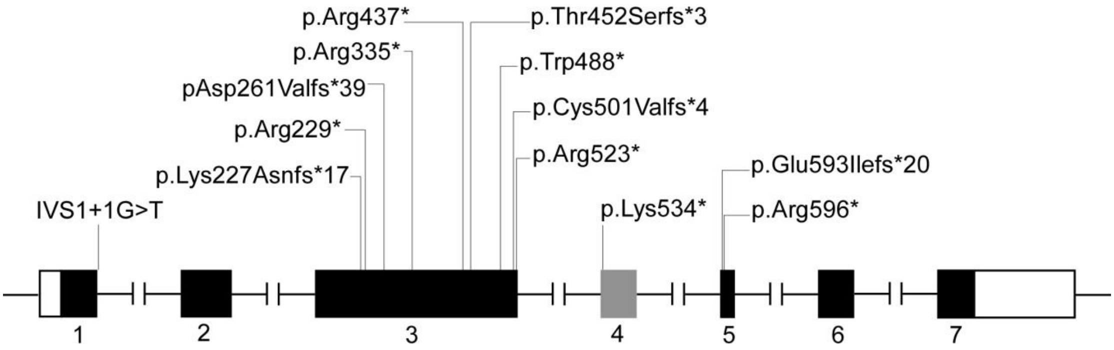

Supplementary Table S1: *FAM161A* mutations reported worldwide (based on NM_001201543)

#	Mutation Name	Location	Number of patients	Reference
1	c.183+1G>T (IVS1+1G>T)	Intron 1	1 compound heterozygous	[1]
2	c.678_681del, p.Lys227Asnfs*17	Exon 3	1 homozygous	[2]
3	c.685C>T, p.Arg229*	Exon 3	1 homozygous	[3]
4	c.728del, p.Asp261Valfs*39	Exon 3	1 homozygous	[2]
5	c.1003C>T, p.Arg335*<br>(reported as c.1105C>T by [4])	Exon 3	2 homozygous	[5]
			3 homozygous	[4]
6	c.1309A>T, p.Arg437*	Exon 3	3 homozygous	[3]
			5 homozygous +<br>3 compound heterozygous	[1]
			6 homozygous	[6]
			1 homozygous +<br>1 compound heterozygous	[7]
			1 compound heterozygous	[8]
			1 compound heterozygous	[9]
7	c.1355_1356del, p.Thr452Serfs*3	Exon 3	26 homozygous +<br>5 compound heterozygous	[10]
			8 homozygous	[11]
			2 homozygous	[12]
8	c.1464G>A, p.Trp488*	Exon 3	1 homozygous	[7]
9	c.1501del, p.Cys501Valfs*4	Exon 3	1 compound heterozygous	[1]
			1 homozygous	[13]
			1 compound heterozygous	[8]
			1 homozygous	[9]
10	c.1567C>T, p.Arg523*	Exon 3	9 homozygous<br>5 compound heterozygous	[10]
			1 compound heterozygous	[1]
			1 compound heterozygous	[7]
			1 homozygous +<br>1 compound heterozygous	[9]
11	c.1600A>T, p.Lys534*	Exon 4	3 homozygous	[14]

12	c.1777_1778del, p.Glu593Ilefs*20 (reported as: p.R592FsX2)	Exon 5	5 homozygous	[15]
13	c.1786C>T, p.Arg596*	Exon 5	2 homozygous	[10]

References:

1 Van Schil, K. *et al.* A Nonsense Mutation in FAM161A Is a Recurrent Founder Allele in Dutch and Belgian Individuals With Autosomal Recessive Retinitis Pigmentosa. *Invest Ophthalmol Vis Sci* **56**, 7418-7426, doi:10.1167/iovs.15-17920 (2015).

2 Carss, K. J. *et al.* Comprehensive Rare Variant Analysis via Whole-Genome Sequencing to Determine the Molecular Pathology of Inherited Retinal Disease. *American journal of human genetics* **100**, 75-90, doi:10.1016/j.ajhg.2016.12.003 (2017).

3 Langmann, T. *et al.* Nonsense mutations in FAM161A cause RP28-associated recessive retinitis pigmentosa. *Am J Hum Genet* **87**, 376-381 (2010).

4 Duncan, J. L. *et al.* Ocular Phenotype of a Family with FAM161A-associated Retinal Degeneration. *Ophthalmic genetics*, 1-9, doi:10.3109/13816810.2014.929716 (2015).

5 Zobor, D., Balousha, G., Baumann, B. & Wissinger, B. Homozygosity mapping reveals new nonsense mutation in the FAM161A gene causing autosomal recessive retinitis pigmentosa in a Palestinian family. *Molecular vision* **20**, 178-182 (2014).

6 Rose, A. M. *et al.* Diverse clinical phenotypes associated with a nonsense mutation in FAM161A. *Eye* **29**, 1226-1232, doi:10.1038/eye.2015.93 (2015).

7 Ellingford, J. M. *et al.* Molecular findings from 537 individuals with inherited retinal disease. *Journal of medical genetics* **53**, 761-767, doi:10.1136/jmedgenet-2016-103837 (2016).

8 Wang, J. *et al.* Dependable and efficient clinical utility of target capture-based deep sequencing in molecular diagnosis of retinitis pigmentosa. *Investigative ophthalmology & visual science* **55**, 6213-6223, doi:10.1167/iovs.14-14936 (2014).

9 Jespersgaard, C. *et al.* Molecular genetic analysis using targeted NGS analysis of 677 individuals with retinal dystrophy. *Scientific reports* **9**, 1219, doi:10.1038/s41598-018-38007-2 (2019).

10 Bandah-Rozenfeld, D. *et al.* Homozygosity mapping reveals null mutations in FAM161A as a cause of autosomal-recessive retinitis pigmentosa. *Am J Hum Genet* **87**, 382-391 (2010).

11 Venturini, G. *et al.* Molecular genetics of FAM161A in North American patients with early-onset retinitis pigmentosa. *PLoS One* **9**, e92479, doi:10.1371/journal.pone.0092479 (2014).

12 Beryozkin, A. *et al.* Whole Exome Sequencing Reveals Mutations in Known Retinal Disease Genes in 33 out of 68 Israeli Families with Inherited Retinopathies. *Scientific reports* **5**, 13187, doi:10.1038/srep13187 (2015).

13 Glockle, N. *et al.* Panel-based next generation sequencing as a reliable and efficient technique to detect mutations in unselected patients with retinal dystrophies. *European journal of human genetics : EJHG* **22**, 99-104, doi:10.1038/ejhg.2013.72 (2014).

14 Maranhao, B. *et al.* Investigating the Molecular Basis of Retinal Degeneration in a Familial Cohort of Pakistani Decent by Exome Sequencing. *PloS one* **10**, e0136561, doi:10.1371/journal.pone.0136561 (2015).

15 Zhou, Y. *et al.* Whole-exome sequencing reveals a novel frameshift mutation in the FAM161A gene causing autosomal recessive retinitis pigmentosa in the Indian population. *Journal of human genetics* **60**, 625-630, doi:10.1038/jhg.2015.92 (2015).

Supplementary Table S2: Clinical parameters of patients who participated in the study

Patient number (age)	Mutation 1	Mutation 2	Age of disease onset	Visual Acuity as Snellen ratio (age if differs from 1st column)[b]	Refraction In diopters (age if differs from 1st column)[c]	Cone flicker 30Hz (µV;msec)[d]	Mixed cone-rod response a wave: b wave (µV)[d]	Rod response (µV)[d]	ERM	Cataract (age at surgery, if performed)
MOL0053-2 (30)[a]<br>MOL0053-3 (44)	p.Arg523*<br>p.Arg523*	p.Arg523*<br>p.Arg523*	18<br>4	0.4<br>0.34	-6.50<br>-7.00	ND<br>NA	ND<br>NA	ND<br>NA	Yes<br>Yes	Yes<br>Yes
MOL0100-1 (10)[a]<br>MOL0100-2 (10)[a]	p.Thr452Serfs*3<br>p.Thr452Serfs*3	p.Thr452Serfs*3<br>p.Thr452Serfs*3	10<br>10	1<br>0.9	-3.25<br>-6.00	ND<br>ND	ND<br>SR	ND<br>ND	Yes<br>Yes	No<br>No
MOL0139-1 (10)[a]<br>MOL0139-2 (43)[a]	p.Thr452Serfs*3<br>p.Thr452Serfs*3	p.Thr452Serfs*3<br>p.Thr452Serfs*3	Childhood<br>NA	0.83<br>0.12	NA<br>NA	33;39<br>SR	80:258<br>SR	216<br>SR	Yes<br>NA	No<br>NA
MOL0195-1 (29)[a]	p.Thr452Serfs*3	p.Arg523*	Teen years	1.25	-6.00	ND	ND	ND	Yes[e]	Yes (37)
MOL0228-1 (11)<br>MOL0228-2 (27)<br>MOL0228-3 (10)	p.Thr452Serfs*3<br>p.Thr452Serfs*3<br>p.Thr452Serfs*3	p.Thr452Serfs*3<br>p.Thr452Serfs*3<br>p.Thr452Serfs*3	11<br>Teen years<br>10	0.63<br>0.45<br>0.5	High myopia<br>-3.00<br>-3.25	ND<br>ND<br>26;32	ND<br>ND<br>55:110	ND<br>ND<br>ND	NA<br>NA<br>Yes	Yes<br>Yes (33)<br>Yes
MOL0276-1 (41)[a]<br>MOL0276-2 (51)[a]	p.Arg523*<br>p.Arg523*	p.Arg523*<br>p.Arg523*	NA<br>NA	0.9<br>0.05	NA<br>-4.75	27;31<br>ND	95:154<br>ND	109<br>ND	Yes<br>Yes	NA<br>Yes
MOL0284-1 (38)[a]	p.Thr452Serfs*3	p.Thr452Serfs*3	2	0.29	-12.00	ND	ND	ND	Yes	Yes (50)
MOL0286-1 (59)[a]	p.Thr452Serfs*3	p.Thr452Serfs*3	3	1.0	-1.75	22;35	67:154	65	Yes	Yes
MOL0303-1 (19)[a]	p.Thr452Serfs*3	p.Arg523*	18	1.0	NA	ND	ND	ND	NA	Yes
MOL0313-1 (35)	p.Thr452Serfs*3	p.Thr452Serfs*3	14	0.14	-6.00	ND	ND	ND	Yes	Yes
MOL0352-1 (78)	p.Thr452Serfs*3	p.Thr452Serfs*3	NA	NA	NA	NA	NA	NA	NA	NA
MOL0446-1 (28)[a]	p.Thr452Serfs*3	p.Arg523*	Childhood	0.65	-9.00	ND	ND	ND	Yes	Yes
MOL0453-1 (43)	p.Thr452Serfs*3	p.Thr452Serfs*3	NA	0.4	-3.75	ND	ND	ND	NA	NA

MOL0526-1 (16) [a]<br>MOL0526-3 (17) [a]	p.Thr452Serfs*3<br>p.Thr452Serfs*3	p.Thr452Serfs*3<br>p.Thr452Serfs*3	Childhood<br>Childhood	0.9<br>1.0	NA<br>NA	ND<br>ND	ND<br>ND	ND<br>ND	Yes[e]<br>Yes	No<br>No
MOL0570-1 (52) [a]	p.Thr452Serfs*3	p.Thr452Serfs*3	Teen years	0.29	-1.75	ND	ND	ND	No	No
MOL0672-1 (41) [a]	p.Thr452Serfs*3	p.Thr452Serfs*3	Childhood	0.85	-12.00	ND	ND	ND	Yes	Yes
MOL0696-1 (29) [a]<br>MOL0696-2 (60) [a]	p.Thr452Serfs*3<br>p.Thr452Serfs*3	p.Thr452Serfs*3<br>p.Thr452Serfs*3	Teen years<br>17	0.8<br>0.01	-7.75 (29)<br>-9.25 (62)	ND<br>ND	ND<br>ND	ND<br>ND	Yes<br>Yes	Yes<br>No
MOL0711-1 (60)<br>MOL0711-2 (64)	p.Arg523*<br>p.Arg523*	p.Arg523*<br>p.Arg523*	Teen years<br>NA	0.05<br>NA	NA<br>NA	ND<br>NA	ND<br>NA	ND<br>NA	NA<br>NA	NA<br>NA
MOL0740-1 (27) [a]<br>MOL0740-2 (19) [a]	p.Thr452Serfs*3<br>p.Thr452Serfs*3	p.Thr452Serfs*3<br>p.Thr452Serfs*3	22<br>Teen years	0.82<br>0.7	-10.25 (33)<br>NA	ND<br>ND	ND<br>ND	ND<br>ND	Yes<br>Yes	No<br>NA
MOL0766-1 (32) [a]<br>MOL0766-2 (39) [a]<br>MOL0766-3 (56)	p.Thr452Serfs*3<br>p.Thr452Serfs*3<br>p.Thr452Serfs*3	p.Thr452Serfs*3<br>p.Thr452Serfs*3<br>p.Thr452Serfs*3	Childhood<br>Childhood<br>NA	1.0<br>0.815<br>0.0001	-7.75<br>-7.25 (33)<br>NA	ND<br>ND<br>ND	ND<br>ND<br>ND	ND<br>ND<br>ND	Yes<br>Yes<br>NA	Yes (42)<br>Yes<br>Yes (57)
MOL0777-1 (7) [a]	p.Arg596*	p.Arg596*	Childhood	0.55	NA	ND	ND	ND	NA	NA
MOL0784-1 (25) [a]<br>MOL0784-2 (25)	p.Arg523*<br>p.Arg523*	p.Arg523*<br>p.Arg523*	Childhood<br>8	0.66<br>0.15	-12.00 (30)<br>-5.25 (49)	ND<br>ND	ND<br>ND	ND<br>ND	Yes<br>Yes	Yes (43)<br>Yes (45)
MOL0786-1 (9) [a]	p.Thr452Serfs*3	p.Thr452Serfs*3	3.5	0.66	-9.00	14:35	SR	SR	Yes	No
MOL0870-2 (48)<br>MOL0870-3 (52)	p.Arg523*<br>p.Arg523*	p.Arg523*<br>p.Arg523*	NA<br>NA	0.07<br>0.11	NA<br>NA	ND<br>ND	ND<br>ND	ND<br>ND	NA<br>NA	NA<br>NA
MOL0878-1 (40)	p.Thr452Serfs*3	p.His441Profs*15	16.5	0.9	-3.00	NA	NA	NA	Yes	Yes (21)
MOL0919-1 (38)	p.Thr452Serfs*3	p.Arg523*	6	0.2	-9.00	ND	ND	ND	Yes	Yes
MOL0935-1 (68)<br>MOL0935-2 (28)<br>MOL0935-3 (31)	p.Thr452Serfs*3<br>p.Thr452Serfs*3<br>p.Thr452Serfs*3	p.Thr452Serfs*3<br>p.Thr452Serfs*3<br>p.Thr452Serfs*3	NA<br>30<br>NA	0.001<br>0.6<br>0.13	-6.25<br>-5.50<br>-6.75	ND<br>SR<br>ND	ND<br>SR<br>ND	ND<br>SR<br>ND	NA<br>Yes<br>NA	NA<br>Yes<br>NA
MOL0952-1 (32)	p.Thr452Serfs*3	p.Thr452Serfs*3	25	0.66	-7.25	SR	SR	SR	Yes	Yes
MOL0969-1 (53)<br>MOL0969-2 (65)<br>MOL0969-3 (62)	p.Thr452Serfs*3<br>p.Thr452Serfs*3<br>p.Thr452Serfs*3	p.Thr452Serfs*3<br>p.Thr452Serfs*3<br>p.Thr452Serfs*3	30<br>NA<br>23	0.85<br>NA<br>0.0001	NA<br>NA<br>NA	NA<br>NA<br>ND	NA<br>NA<br>ND	NA<br>NA<br>ND	Yes<br>NA<br>Yes	Yes<br>NA<br>Yes
MOL0978-1 (45)	p.Thr452Serfs*3	p.Thr452Serfs*3	NA	NA	NA	ND	ND	ND	No	Yes
MOL1024-1 (62)	p.Thr452Serfs*3	p.Thr452Serfs*3	13	0.0001	High myopia	NA	NA	NA	Yes	Yes
MOL1027-1 (31)	p.Thr452Serfs*3	p.Thr452Serfs*3	Teen years	0.9	-12.00	7:34	27;52	ND	Yes	Yes
MOL1039-1 (15)	p.Thr452Serfs*3	p.Thr452Serfs*3	11	0.57	-11.25	ND	ND	ND	NA	NA
MOL1043-1 (40)	p.Thr452Serfs*3	p.Thr452Serfs*3	27	1.0	-3.25	SR	SR	SR	Yes	Yes
MOL1053-1 (58)<br>MOL1053-2 (50)	p.Thr452Serfs*3<br>p.Thr452Serfs*3	p.Arg523*<br>p.Arg523*	Teen years<br>NA	0.12<br>NA	-5.50<br>NA	5:27<br>NA	ND<br>NA	ND<br>NA	Yes<br>NA	Yes (52)<br>NA

MOL1073-1 (43)<br>MOL1073-2<br>MOL1073-3	p.Thr452Serfs*3<br>p.Thr452Serfs*3<br>p.Thr452Serfs*3	p.Thr452Serfs*3<br>p.Thr452Serfs*3<br>p.Thr452Serfs*3	30<br>NA<br>NA	0.63<br>NA<br>NA	-4.50<br>NA<br>NA	NA<br>NA<br>NA	NA<br>NA<br>NA	NA<br>NA<br>NA	Yes<br>NA<br>NA	Yes (34)<br>NA<br>NA
MOL1160-1 (61)	p.Thr452Serfs*3	p.Thr452Serfs*3	18	0.2	-3.75	ND	ND	ND	Yes	Yes (65)
MOL1200-1 (29)	p.Thr452Serfs*3	p.Thr452Serfs*3	30	1.0	NA	ND	ND	ND	Yes	Yes
MOL1209-1 (18)	p.Thr452Serfs*3	p.Thr452Serfs*3	10	0.8	NA	10:26	3;36	ND	Yes	No
MOL1221-1	p.Arg523*	p.Arg523*	NA	NA	NA	NA	NA	NA	NA	NA
MOL1289-1 (9)	p.Thr452Serfs*3	p.Thr452Serfs*3	9	0.5	-9.75	ND	ND	ND	Yes	NA
MOL1294-1 (31)	p.Thr452Serfs*3	p.Thr452Serfs*3	NA	1.0	NA	NA	NA	NA	NA	NA
MOL1300-1 (9)<br>MOL1300-2 (19)<br>MOL1300-3 (9)	p.Arg523*<br>p.Arg523*<br>p.Arg523*	p.Arg523*<br>p.Arg523*<br>p.Arg523*	Childhood<br>Childhood<br>Childhood	0.45<br>0.1<br>0.43	-1.50<br>-4.00<br>-1.25	ND<br>ND<br>ND	ND<br>ND<br>ND	ND<br>ND<br>ND	NA<br>NA<br>NA	NA<br>NA<br>NA
MOL1335-1<br>MOL1335-2	p.Thr452Serfs*3<br>p.Thr452Serfs*3	p.Thr452Serfs*3<br>p.Thr452Serfs*3	NA<br>NA	NA<br>NA	High myopia<br>NA	NA<br>NA	NA<br>NA	NA<br>NA	NA<br>NA	Yes<br>NA
MOL1350-1 (69)	p.Thr452Serfs*3	p.Thr452Serfs*3	NA	0.0001	NA	ND	ND	ND	Yes	NA
MOL1365-1 (82)	p.Thr452Serfs*3	p.Thr452Serfs*3	NA	NA	NA	NA	NA	NA	NA	NA
MOL1380-1 (47)	p.Thr452Serfs*3	p.Thr452Serfs*3	Childhood	0.33	-4.00	ND	ND	ND	Yes	Yes (63)
MOL1387-1 (17)	p.Thr452Serfs*3	p.Thr452Serfs*3	Childhood	0.65	NA	9;40	38:58	ND	Yes	NA
MOL1392-1 (43)	p.Thr452Serfs*3	p.Thr452Serfs*3	NA	0.8	NA	13;36	ND	ND	Yes	Yes
MOL1426-1 (40)	p.Thr452Serfs*3	p.Arg523*	18	0.3	-3.50	21;31	27:41	ND	Yes	Yes
MOL1504-1 (20)	p.Thr452Serfs*3	p.Thr452Serfs*3	6	1	-4.75	10;43	29:77	64	Yes	NA
MOL1545-1 (19)<br>MOL1545-2 (14)<br>MOL1545-4 (7)	p.Arg523*<br>p.Arg523*<br>p.Arg523*	p.Arg523*<br>p.Arg523*<br>p.Arg523*	Childhood<br>Childhood<br>5	0.45<br>0.44<br>0.5	-5.75<br>-7.75<br>-6.75	ND<br>ND<br>SR	ND<br>ND<br>SR	ND<br>ND<br>SR	No<br>No<br>No	No<br>No<br>No
MOL1649-1 (62)	p.Thr452Serfs*3	p.Thr452Serfs*3	60	0.8	-0.75	11;40	ND	ND	Yes	Yes
MOL1666-1 (45)<br>MOL1666-2 (38)	p.Thr452Serfs*3<br>p.Thr452Serfs*3	p.Thr452Serfs*3<br>p.Thr452Serfs*3	NA<br>NA	NA<br>NA	NA<br>NA	ND<br>ND	ND<br>ND	ND<br>ND	NA<br>NA	NA<br>NA
MOL1668-1 (5)	p.Thr452Serfs*3	p.Arg523*	NA	NA	NA	14;33	ND	ND	NA	NA
MOL1750-1 (15)	p.Thr452Serfs*3	p.Thr452Serfs*3	Childhood	0.8	-7.50	ND	ND	ND	Yes	No
MOL1765-1 (20)	p.Thr452Serfs*3	p.Thr452Serfs*3	20	0.8	NA	27;44	ND	ND	Yes	No
MOL1773-1 (39)	p.Thr452Serfs*3	p.Arg523*	Childhood	0.8	NA	ND	ND	ND	Yes	Yes
MOL1779-1 (18)	p.Thr452Serfs*3	p.Thr452Serfs*3	NA	0.8	NA	ND	ND	ND	NA	NA
MOL1793-1 (62)	p.Thr452Serfs*3	p.Thr452Serfs*3	7	0.06	-8.00	ND	ND	ND	Yes	Yes
MOL1831-1 (86)	p.Thr452Serfs*3	p.Thr452Serfs*3	NA	0.0001	High myopia	ND	ND	ND	Yes	Yes

MOL1833-1 (30)	p.Thr452Serfs*3	p.Arg523*	10	0.45	-8.00	ND	ND	ND	NA	NA
TB21-R28 (24) [a]	p.Thr452Serfs*3	p.Thr452Serfs*3	12	0.001	NA	30;28	ND	ND	Yes	Yes
TB21-R27 (34)	p.Thr452Serfs*3	p.Thr452Serfs*3	34	0.31	-9.65	ND	ND	ND	NA	Yes
TB21-R29	p.Thr452Serfs*3	p.Thr452Serfs*3	NA	NA	NA	NA	NA	NA	NA	NA
TB21-R20 (63) [a]	p.Thr452Serfs*3	p.Thr452Serfs*3	50	0.3	NA	ND	ND	ND	Yes[e]	Yes
TB65-U52 (27)	p.Thr452Serfs*3	p.Thr452Serfs*3	Childhood	0.8	NA	ND	ND	ND	Yes	Yes
TB65-U61	p.Thr452Serfs*3	p.Thr452Serfs*3	NA	NA	NA	NA	NA	NA	NA	NA
TB112-835522 (64)	p.Thr452Serfs*3	p.Thr452Serfs*3	40	0.001	-4.50	ND	ND	ND	Yes	Yes (68)
TB155-R417 (52)	p.Thr452Serfs*3	p.Thr452Serfs*3	20	0.05	NA	9;39	22:16	ND	Yes	Yes
TB190-R476 (52)	p.Thr452Serfs*3	p.Thr452Serfs*3	45	0.28	NA	SR	SR	SR	Yes[e]	Yes
TB190-R477 (24)	p.Thr452Serfs*3	p.Thr452Serfs*3	20	NA	NA	ND	ND	ND	Yes	No
TB195-R492 (65)	p.Thr452Serfs*3	p.Thr452Serfs*3	17	NA	NA	NA	NA	NA	NA	NA
TB205-R515 (56)	p.Thr452Serfs*3	p.Thr452Serfs*3	40	NA	NA	NA	NA	NA	NA	NA
TB250-R582 (33)	p.Thr452Serfs*3	p.Arg523*	20	0.58	-8.00	ND	ND	ND	Yes	No
TB332-R710 (39)	p.Thr452Serfs*3	p.Thr452Serfs*3	29	NA	NA	ND	ND	ND	NA	NA
TB346-R732 (51)	p.Thr452Serfs*3	p.Thr452Serfs*3	Childhood	NA	NA	NA	NA	NA	NA	NA
TB370-R760 (44)	p.Thr452Serfs*3	p.Thr452Serfs*3	24	0.6	-6.00	9;41	44:117	16	Yes	Yes
TB379-R777 (58)	p.Thr452Serfs*3	p.Thr452Serfs*3	Childhood	0.0001	NA	ND	ND	ND	Yes	Yes (57)
TB490-R940 (56)	p.Thr452Serfs*3	p.Thr452Serfs*3	NA	NA	NA	51;38	81:90	ND	Yes	NA
TB525-R994 (17)	p.Thr452Serfs*3	p.Arg523*	8	0.4	-15.00	ND	ND	ND	No	No
TB525-R995 (21)	p.Thr452Serfs*3	p.Arg523*	14	0.7	-10.00	ND	ND	ND	No	Yes
TB539-R1015 (69)	p.Thr452Serfs*3	p.Thr452Serfs*3	20	0.35	-3.00	ND	ND	ND	Yes	Yes
TB712-R1292 (31)	p.Thr452Serfs*3	p.Thr452Serfs*3	7	0.66	-5.25	11;37	SR	ND	Yes	No
TB790-R1390 (32)	p.Thr452Serfs*3	p.Thr452Serfs*3	32	0.75	-2.75	ND	ND	ND	Yes	No
TB836-R1466 (4)	p.Thr452Serfs*3	p.Thr452Serfs*3	4	0.01	NA	ND	ND	ND	No	No
TB840-R1474 (36)	p.Thr452Serfs*3	p.Thr452Serfs*3	10	0.5	-10.00	ND	ND	ND	Yes	Yes (45)

ND - non-detectable, SR- severely reduced, NA- not available

a - Clinical data regarding these patients were previously published[9]

b - Best corrected visual acuity is presented as an average of the two eyes, in decimal values. In order to provide numerical values for low VA, the following conversions were made: NLP (no light perception) = 0; LP (light perception) = 0.0001; HM (hand movement) = 0.001; FC (finger counting) = 0.01.

c - Refraction is presented as average spherical equivalent of both eyes, in diopters

d - Full field ERG testing performed according to ISCEV standard; average of the values recorded in the two eyes are shown for each of the main stimulus conditions. Limits of normal are as follows: 30Hz Cone Flicker: lower threshold of normal for amplitude-60µV; upper limit for implicit time- 33msec; Mixed cone-rod response- lower threshold of normal for b wave amplitude- 400µV; for a-wave- 100µV; Rod response- lower threshold of normal for amplitude- 200µV.

e - Macular edema

Supplementary Table S3: Statistical analysis of BCVA in patients with *FAM161A* genotypes

**A. Analysis of Covariance including all 3 *FAM161A* genotypes. Tests of Between-Subjects Effects**						
Dependent Variable:	**Average VA Snellen**					
**Source**	**Type III Sum of Squares**	**df**	**Mean Square**	**F**	**Sig.**	
Corrected Model	14.659[a]	5	2.932	42.181	0.000	
Intercept	20.045	1	20.045	288.402	0.000	
Genotype	2.303	2	1.151	16.565	**p<0.001**	
AGE (years)	7.013	1	7.013	100.902	**p<0.001**	
Genotype * AGE (years)	1.700	2	0.850	12.228	**p<0.001**	
Error	25.021	360	0.070			
Total	122.967	366				
**Corrected Total**	39.680	365				
**a. R Squared = .369 (Adjusted R Squared = .361)**						
**B. Analysis of Covariance, comparing homozygotes for Frameshift versus Compound heterozygotes: Tests of Between-Subjects Effects**						
Dependent Variable:	**Average VA Snellen**					
**Source**	**Type III Sum of Squares**	**df**	**Mean Square**	**F**	**Sig.**	

Corrected Model	10.509[a]	3	3.503	46.114	0.000	
Intercept	18.794	1	18.794	247.417	0.000	
Genotype	1.336	1	1.336	17.593	**p<0.001**	
AGE (years)	7.209	1	7.209	94.903	**p<0.001**	
Genotype * AGE (years)	1.660	1	1.660	21.853	**p<0.001**	
Error	22.940	302	0.076			
Total	116.164	306				
Corrected Total	33.449	305				
a. R Squared = .314 (Adjusted R Squared = .307)						

C. Analysis of Covariance, comparing homozygotes Frameshift versus homozygotes Nonsense: Tests of Between-Subjects Effects

Dependent Variable:	**Average VA Snellen**					
**Source**	**Type III Sum of Squares**	**df**	**Mean Square**	**F**	**Sig.**	
Corrected Model	10.216[a]	3	3.405	52.424	0.000	
Intercept	13.647	1	13.647	210.102	0.000	
Genotype	0.735	1	0.735	11.313	**0.001**	
AGE (years)	2.706	1	2.706	41.653	**p<0.001**	
Genotype * AGE (years)	0.004	1	0.004	0.061	**0.805**	

Error	19.876	306	0.065			
Total	102.982	310				
Corrected Total	30.092	309				
a. R Squared = .339 (Adjusted R Squared = .333)						
**D. Analysis of Covariance, comparing Compound heterozygotes versus homozygotes Nonsense: Tests of Between-Subjects Effects**						
Dependent Variable:	**Average VA Snellen**					
**Source**	**Type III Sum of Squares**	**df**	**Mean Square**	**F**	**Sig.**	
Corrected Model	5.924[a]	3	1.975	30.606	0.000	
Intercept	10.400	1	10.400	161.201	0.000	
Genotype	2.294	1	2.294	35.555	**p<0.001**	
AGE (years)	4.633	1	4.633	71.809	**p<0.001**	
Genotype * AGE (years)	1.199	1	1.199	18.590	**p<0.001**	
Error	7.226	112	0.065			
Total	26.788	116				
Corrected Total	13.149	115				
a. R Squared = .450 (Adjusted R Squared = .436)						

**Generalized Linear Model with repeated measurements: Tests of Model Effects**						
Source	Type III					
	Wald Chi-Square	df	Sig.			
(Intercept)	73.401	1	0.000			
Genotype	15.578	2	**p<0.001**			
AGE (years)	46.579	1	**p<0.001**			
Mutation Type * AGE (years)	8.638	2	**0.013**			
Dependent Variable: Average VA Snellen<br>Model: (Intercept), Mutation Type, AGE (years), Mutation Type * AGE (years)						

Supplementary Table S4: Statistical analysis of VF in patients with *FAM161A* mutations versus patients with mutations in other RP-causing genes.

**A. Pearson Correlation:**						
		Visual Field Area (cm2)				
Age	Pearson Correlation	**-0.129**				
	Sig. (2-tailed)	0.185				
	N	108				
**Spearman's rho:**						
		Visual Field Area (cm2)				
Age	Correlation Coefficient	**-0.088**				
	Sig. (2-tailed)	0.366				
	N	108				
**Pearson Correlation: Different genes**						
Gene name			Visual Field Area (cm2)			
MAK	Age	Pearson Correlation	-.724**			
		Sig. (2-tailed)	0.000			
		N	27			

RPGR	Age	Pearson Correlation	-.580*			
		Sig. (2-tailed)	0.048			
		N	12			
DHDDS	Age	Pearson Correlation	**-0.245**			
		Sig. (2-tailed)	0.442			
		N	12			
FAM161A	Age	Pearson Correlation	**0.194**			
		Sig. (2-tailed)	0.149			
		N	57			
**. Correlation is significant at the 0.01 level (2-tailed).<br>*. Correlation is significant at the 0.05 level (2-tailed).						
**B. Spearman's rho: Different genes**						
Gene name			Visual Field Area (cm2)			
MAK	Age	Correlation Coefficient	-.788**			
		Sig. (2-tailed)	0.000			
		N	27			
RPGR	Age	Correlation Coefficient	**-0.453**			

		Sig. (2-tailed)	0.140			
		N	12			
DHDDS	Age	Correlation Coefficient	**0.007**			
		Sig. (2-tailed)	0.983			
		N	12			
FAM161A	Age	Correlation Coefficient	**0.011**			
		Sig. (2-tailed)	0.936			
		N	57			
**. Correlation is significant at the 0.01 level (2-tailed).						
**C. Analysis of Covariance: Tests of Between-Subjects Effects**						
Dependent Variable:	Visual Field Area (cm2)					
Source	Type III Sum of Squares	df	Mean Square	F	Sig.	
Corrected Model	61105.896[a]	7	8729.414	17.846	0.000	
Intercept	26487.945	1	26487.945	54.152	0.000	
Gene	46682.434	3	15560.811	31.812	**p<0.001**	
Age	9090.285	1	9090.285	18.584	**p<0.001**	
Gene * age	29128.015	3	9709.338	19.850	**p<0.001**	

Error	48914.320	100	489.143			
Total	151717.967	108				
Corrected Total	110020.215	107				
a. R Squared = .555 (Adjusted R Squared = .524)						
**D. Analysis of Covariance, FAM161A vs. MAK**						
Dependent Variable:	Visual Field Area (cm2)					
Source	Type III Sum of Squares	df	Mean Square	F	Sig.	
Corrected Model	54104.895[a]	3	18034.965	42.450	0.000	
Intercept	46564.430	1	46564.430	109.602	0.000	
Gene	43283.565	1	43283.565	101.879	**p<0.001**	
Age	21710.042	1	21710.042	51.100	**p<0.001**	
Gene * age	26086.272	1	26086.272	61.401	**p<0.001**	
Error	33988.090	80	424.851			
Total	115112.313	84				
Corrected Total	88092.985	83				
a. R Squared = .614 (Adjusted R Squared = .600)						

**E. Analysis of Covariance, FAM161A vs. RPGR**						
Dependent Variable:	Visual Field Area (cm2)					
Source	Type III Sum of Squares	df	Mean Square	F	Sig.	
Corrected Model	7332.330[a]	3	2444.110	10.390	0.000	
Intercept	7983.718	1	7983.718	33.938	0.000	
Gene	6937.289	1	6937.289	29.490	**p<0.001**	
Age	4379.930	1	4379.930	18.619	**p<0.001**	
Gene * age	5278.146	1	5278.146	22.437	**p<0.001**	
Error	15290.850	65	235.244			
Total	28478.345	69				
a. R Squared = .324 (Adjusted R Squared = .293)						
**F. Analysis of Covariance, FAM161A vs. DHDDS**						
Dependent Variable:	Visual Field Area (cm2)					
Source	Type III Sum of Squares	df	Mean Square	F	Sig.	
Corrected Model	5992.598[a]	3	1997.533	12.756	0.000	
Intercept	1617.701	1	1617.701	10.330	0.002	

Gene	1311.998	1	1311.998	8.378	**0.005**	
Age	223.810	1	223.810	1.429	**0.236**	
Gene * age	397.653	1	397.653	2.539	**0.116**	
Error	10178.794	65	156.597			
Total	24215.869	69				
Corrected Total	16171.392	68				
a. R Squared = .371 (Adjusted R Squared = .342)						

Supplementary Table S5: Sequence of primers used in this study

	Forward	Reverse
Ex 3a	TGAGCATGGTGGCACAAG	GCTGACCTACAAGGCAGAGG
Ex 3b	TGCATCTGTCTTTCTCCCC	TCTTAGAATACCCTCTGACAAAATATC
Ex 4	ATCCCATGTTAAATCTTTGC	GAAAACCAGTGGTCTGGAG
